# Transcriptional Landscape of PARs in Epithelial Malignancies

**DOI:** 10.3390/ijms19113451

**Published:** 2018-11-02

**Authors:** Jeetendra Kumar Nag, Rachel Bar-Shavit

**Affiliations:** Sharett Institute of Oncology, Hadassah-Hebrew University Medical Center, P.O. Box 12000, Jerusalem 91120, Israel; jeetendr.nag@mail.huji.ac.il

**Keywords:** PARs, EGR-1, p53, ERE, ARE, Twist, TEAD4, Sp1, AP-2

## Abstract

G protein-coupled receptors (GPCRs), the largest family of cell receptors, act as important regulators of diverse signaling pathways. Our understanding of the impact of GPCRs in tumors is emerging, yet there is no therapeutic platform based on GPCR driver genes. As cancer progresses, it disrupts normal epithelial organization and maintains the cells outside their normal niche. The dynamic and flexible microenvironment of a tumor contains both soluble and matrix-immobilized proteases that contribute to the process of cancer advancement. An example is the activation of cell surface protease-activated receptors (PARs). Mammalian PARs are a subgroup of GPCRs that form a family of four members, PAR_1–4_, which are uniquely activated by proteases found in the microenvironment. PAR_1_ and PAR_2_ play central roles in tumor biology, and PAR_3_ acts as a coreceptor. The significance of PAR_4_ in neoplasia is just beginning to emerge. PAR_1_ has been shown to be overexpressed in malignant epithelia, in direct correlation with tumor aggressiveness, but there is no expression in normal epithelium. In this review, the involvement of key transcription factors such as Egr1, p53, Twist, AP2, and Sp1 that control PAR_1_ expression levels specifically, as well as hormone transcriptional regulation by both estrogen receptors (ER) and androgen receptors (AR) are discussed. The cloning of the human protease-activated receptor 2; *Par2* (*hPar2*) promoter region and transcriptional regulation of estrogen (E_2_) via binding of the E_2_–ER complex to estrogen response elements (ERE) are shown. In addition, evidence that TEA domain 4 (TEAD_4_) motifs are present within the *hPar2* promoter is presented since the YAP oncogene, which plays a central part in tumor etiology, acts via the TEAD_4_ transcription factor. As of now, no information is available on regulation of the *hPar3* promoter. With regard to *hPar4*, only data showing CpG methylation promoter regulation is available. Characterization of the PAR transcriptional landscape may identify powerful targets for cancer therapies.

## 1. Introduction

**Figure 1:**
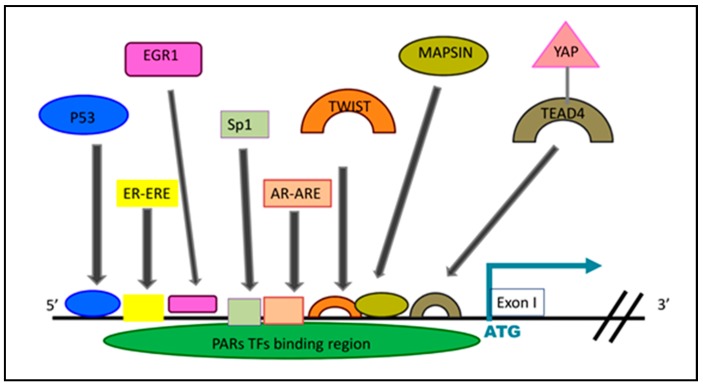
Schematic illustration of the PAR promoter region and allocated TF, focusing on PAR_1_ and PAR_2_ promoters with related TFs and putative TF motifs.

G protein-coupled receptors (GPCRs) are the largest family of cell surface receptors and they are involved in a wide array of physiological processes, yet their role in cancer etiology is poorly addressed [1,2,3]. Mammalian protease-activated receptors (PARs), a subgroup of GPCRs, is a family of four members that are activated by both soluble and matrix-immobilized proteases present in the active and flexible tumor microenvironment. Proteolytic activation of PARs contributes immensely to cancer progression. PAR_1_ and PAR_2_ are known to play a central part in tumor biology [4,5,6,7,8,9,10]. The molecular machinery associated with transition of a primary tumor from a local disease to metastatic dissemination is the center of intense studies and an ongoing challenge. PAR_1_ was shown to be overexpressed in direct correlation to the aggressiveness of carcinomas, compared with no expression in normal epithelia. The *hPar1* mRNA level was shown to be high in aggressive tumors utilizing a panel of tissue biopsy specimens and cell lines accompanied by in situ hybridization and reverse transcription-PCR (RT-PCR) analyses [9]. In parallel, fluorescence in situ chromosome hybridization assays performed on cells of high (e.g., CL1) and low (e.g., LNCaP) metastatic potential showed that the *hPar1* gene copy number remains unchanged regardless of the *hPar1* expression level, indicating that overexpression of *hPar1* does not stem from gene amplification. Consequently, *hPar1* transcription rates and mRNA stability were evaluated to determine the elevation rate of *hPar1* mRNA levels. To analyze the stability of *hPar1* mRNA, cells were treated with the transcription inhibitor 5,6-dichloro-1-β-d-ribofuranosyl benzimidazol (DRB). At various time points, mRNA was extracted and the level of *hPar1* mRNA was analyzed by both Northern blotting and real-time PCR to determine levels of *hPar1* mRNA. Degradation rates for *hPar1* mRNA were similar regardless of whether RNA came from cells with high (e.g., CL1 or PC3; data not shown) or low (e.g., LNCaP) *hPar1* expression levels [9]. In contrast, by applying a nuclear run-on assay to detect transcript elongation rates, a markedly enhanced *hPar1* transcription rate was observed in the highly metastatic PC3 cells, which express high *hPar1* levels compared with LNCaP of low metastatic potential where there are low levels of *hPar1* expression [9]. Hence, it was concluded that increased *hPar1* RNA levels in the malignant cells are primarily due to increased *hPar1* transcription. This outcome guided us to center our focus and study the transcription factor (TF) landscape associated with PAR overexpression. Significantly, master TFs are conserved throughout evolution in coordinating transcriptional gene regulation networks functioning via binding to specific short sequence arrays (“motifs”) in matching promoter regions to control the transcriptional expression of a panel of target genes in a plethora of pathological and physiological functions (Figure 1). 

The idea of a rigid hierarchical stem cell niche organization in a tumor has been challenged, suggesting that within a heterogeneous cancer cell population, targeting and identifying the stem cell compartment is the main task ahead. PAR_1&2_ play a central role in epithelial tumor advancement and are potent inducers of the canonical Wnt/β-catenin stabilization path, a core process both in developmental and tumor progression pathways [10,11]. While PAR_3_ is a co-receptor, PAR_4_ (named F2RL3) has emerged as a potent stem cell marker out of a wide panel of GPCR-induced stem-cell sphere formation candidates identified with high-throughput RNA sequencing [12].

In the present review we outline and center on relevant TFs that regulate the expression levels of PAR family members (mainly PAR_1_, and partially PAR_2_ and PAR_4_) in epithelial malignancies. In this respect, we discuss up-to-date knowledge on transcriptional controls including biochemical and structural aspects. While PAR_1_ is the prototype member, overexpressed directly with the tumor aggressiveness, we address also hormone regulation of PAR_1_ and PAR_2_, as also YAP regulation by TEA domain 4 (TEAD4) on PAR_2_. The regulation of *hPar4* by CpG methylation is discussed as well. Finally, we provide data by searching online platform of patient data available at Gene Expression Profiling Interactive Analysis (GEPIA, http://gepia.cancer-pku.cn), on expression levels of TFs in normal and pathological epithelia.

## 2. Egr-1 Binds to *hPar1* Promoter and Increases PAR_1_ Expression

The promoter of the PAR_1_ gene is comprised of multiple alleged consensus elements for a plethora of TFs. As a consequence, these TFs are physically associated with the promoter region and regulate PAR_1_ expression levels. However, sequence-constructed methods as such are insufficient to describe the complete contact specificities of TF-DNA in vivo. To improve the specificity of candidate gene likelihoods, it is necessary to integrate appropriate context expression significance between TF and downstream genes as well as chromatin structural aspects. Chromatin immunoprecipitation (ChIP) analysis of the *hPar1* promoter area along with luciferase-promoter activity aided in revealing the up-to-date part of the explicit transcription factors involved in PAR-induced epithelial cancer growth and progression [9,13,14,15]. As such, analysis of the *hPar1* promoter genomic sequence (accession number U63331) reveals a potential Early Growth Response-1 (Egr-1) motif located between −354 and −335 bp3 [9].

Although a direct association between Egr-1 and the *hPar1* promoter region was shown, the possibility that Egr-1 binds initially with Sp1 to form an Sp1/Egr-1 complex, as shown in the control of hepatocyte growth factor levels [16], cannot be ruled out. However, we have presented firm evidence for the functional involvement of Egr-1 in increased *hPar1* expression in prostate carcinoma [9]. Egr-1 is a zinc finger TF that centrally acts in regulating cell growth, proliferation, differentiation, and apoptosis [17,18,19,20,21]. Egr-1 binds to GC-rich consensus DNA motifs residing in regulatory areas, thereby controlling the transcription of target genes. Interestingly, not only does it bind to the promoter region of genes that act as oncogenes, it also emerges as performing a role in determining microRNA (miRNA) levels in the context of tumor biology. In recent years, one of the main cornerstones for advancing our understanding of the central mechanisms of gene control has been findings relating to microRNAs/miRs. These are small ≈22-nucleotide (nt) noncoding RNAs [22,23,24,25] generated from bulky primary miRNAs (pri-miRNA) that are processed to ≈70-nt precursors (pre-miRNA) and then to the final form by endonucleases [26,27,28,29]. Approximately 30% of all protein-coding genes are anticipated to be processed by miRNAs [28]. miRNAs retain various tasks in numerous biological and pathological processes, comprising the regulation of cell proliferation, differentiation, and apoptosis. Abnormal expression and dysregulation of miRNAs add to angiogenesis, tumorigenesis, and metastasis [27,30,31]. 

Our current understanding is that miRNAs can function as either oncogenes or tumor suppressors [31,32]. Because of the transient and dynamic nature of pri-miRNAs, owing to their rapid processing, the transcription start site (TSS) design approach relying on RNA evaluations, as in the case of miRNA, is challenging. Recent studies have found chromatin signatures that can be utilized for promoter regulatory site identification. Zhang et al. [31] discovered discrete histone alteration arrangements for promoters and enhancers, and showed the likelihood of their use in determining original control elements with chromatin immunoprecipitation (ChIP)-chip screens. Additionally, transcriptionally active genes show nucleosome reduction in the 100- to 130-base-pair (bp) gap near their TSS [33,34,35]. For example, it has been demonstrated that Egr-1 controls the transcription of miR-20b in breast cancer. Ionizing radiation (IR) elicits an increase in miR20b and Egr-1 expression in breast cancer. In fact, miR-20b targets the tumor suppressor genes BRCA1 and PTEN and therefore these tumor suppressors are silenced. Subsequently, miR-20b functions as an oncomiR by targeting tumor suppressor genes and tilting the balance towards oncogenicity. Egr-1 facilitates the transcription of miR-20b, thereby inducing the growth and progression of breast cancer. This suggests that both Egr-1 and miR-20b are potent targets for cancer therapy [36]. 

Alternatively, Egr-1 may also act to inhibit cancer progression by targeting, for instance, miR-203a, which functions as an anti-oncogene. It can induce expression of miR-203a and indirectly inhibit the expression of HOXD3 transcription factor in hepatocellular carcinoma (HCC) via miR-203a, causing attenuation of HCC progression [37]. HOXD3 silencing and/or degradation substantially decreased HCC cell migration, invasion, and angiogenesis. Thus, it might serve as a likely future therapeutic approach for HCC. 

Aspects of chromatin architecture and structure should also be considered when discussing the likelihood of TF accessibility and association with target gene promoters. Chromatin looping and participation of histone acetyltransferases p300 and CREB binding protein (CBP) are necessary for an open chromatin design and are the required components for bridging the gap between target gene-promoters and a new regulatory element termed enhancer RNA. Enhancers are another group of important control components of the genome that contribute to appropriate instigation of gene regulatory sites via creation of chromosomal coils [38]. An example of the participation of enhancer regulation is the novel transcriptional enhancer (named eRNA) for the heparanase (*HSPE*) gene. This is a well-known endo-β-d-glucuronidase that plays an important role since it cleaves and degrades the basement membrane component heparan sulfate, thereby promoting tumor invasion and metastasis. HPSE eRNA associates with the heterogeneous nuclear ribonucleoprotein U (hnRNPU) to facilitate its contact with p300, consequently giving rise to chromatin looping between the super enhancer, eRNA, and the HPSE regulatory region. Recruitment of Egr-1 causes elevated expression and function of HPSE. Generally, this indicates the crucial roles of the axis: eRNA/hnRNPU/p300/EGR1/HPSE in tumor development [39].

Increasing evidence suggests that Egr-1 stimulation may act as a master alteration in many pathological processes, including cardiovascular diseases and cancers. Egr-1 has been designated in the development of a spectrum of epithelia-derived tumors such as breast, prostate, colon, and esophageal cancers [40,41,42,43,44]. Enhanced Egr-1 in esophageal cancer plays a significant part in facilitating advancement-associated oncogene/CXC chemokine receptor 2 proliferative signaling [42]. Egr-1 is upregulated in primary human prostate carcinomas [41,42,43] and in numerous downstream Egr-1 genes (e.g., transforming growth factor β1, insulin-like growth factor II, and platelet-derived growth factor A-chain), that have been connected to prostate cancer [44]. The silencing of Egr-1 inhibits the proliferation of prostate cancer cell and growth in the transgenic adenocarcinoma prostate of mice [45].

## 3. The Interrelations of *hPar1* and p53

In the last 20 years it has become evident that the tumor suppressor p53 and its tumor-associated mutants (*mt*) p53 play distinctly different roles. Whereas wild-type (*wt*) p53 acts in controlling the expression of genes that control a selection of cell-associated procedures comprising apoptosis, cell senescence, and cell cycle checkpoints, the *mt* p53 primarily act as oncogenes endorsing cell survival, invasion, and metastasis. We have demonstrated that *wt* p53 negatively regulates the level of *hPar1* via transcriptional inhibition [13]. 

In a tumor, when *wt* p53 expression is either lost or mutated, the tumor suppressor properties of p53 are lost. More bizarrely, *mt* p53 acquires the “know-how” to provoke tumor aggressiveness, invasion, chemo-resistance, and genomic instability. These properties of *mt* p53 are also known as p53 “gain-of-function” of pro-tumor tasks that are completely independent of *wt* p53 functions [46,47,48,49,50]. The p53 gene presents the greatest range of genetic variation found so far in human tumors, and affects more than 50% of all cancers [51,52]. Frequent mutations in several “hot spots” (among which are: R175, G245, R248, R249, R273, and R282), may provide some hints on the effect of the functions of p53. These mutations are often placed in the DNA-binding area of p53 [53,54]. As such, a collection of the mutations in the DNA binding region indicates its critical role and impact on alterations in the *mt* p53 target genes. Remarkably, mutations in the central region of the protein may also provide clues concerning the significance of the structural folding design of p53. The mutations are divided into two groups; those that are related to architectural aspects (such as R175H, which is unfolded under physiological situations) and those that are located at the DNA binding region. While structural features may account for the gain-of-function of *mt* pro tumor p53, the DNA-binding alterations may suggest that these mutants recognize a specific response element for *mt* p53, permitting their oncogenic function; however, there is no consensus on the sequence of such a response element. 

The best known transcriptional role of *mt* p53 relates to its ability to associate with other transcription factors and modify their target gene levels. For example, the *mt* p53 (p53R175H) induces EGR-1 via physical contact between the *mt* p53-EGR-1 promoter, forming a complex that provides considerable action related to oncogenic gain-of-function [55]. As discussed in the previous paragraph, the transcription factor EGR-1 has been shown to associate with the promoter of *hPar1* in prostate cancer, leading to *hPar1* overexpression and enhancement of invasive properties [9]. In general, *hPar1* levels may be stimulated indirectly by *mt* p53 via the induced EGR-1 level. Another option is association via the NF-Y transcription factor that binds to the CCAAT motif sequence [56], two of which are found within the *hPar1* regulatory area (at −2736 and −2516) to recruit *mt* p53. The acetylase p300 is bound to the *mt* p53-*hPar1* promoter complex, where it helps to “open” the chromatin structure toward transcription [57,58]. Notably, *wt* p53 is a sequence-specific transcription factor that associates with selective response elements and is negatively regulated by Mdm2, an E3 ubiquitin ligase that assigns p53 for ubiquitination and proteasomal degradation. Bioinformatic analysis for the p53 consensus sites [13] showed two motifs between −2936 to −2916 and −1724 to −1704, upstream to the PAR_1_ start-site coding region. ChIP analyses conclusively exhibited direct binding and physical association between p53 and the *hPar1* regulatory area for a delicate fine-tuning of the PAR_1_ oncogenic function in prostate cancer growth and progression. Along with this line of evidence, while there is an inverse association between PAR_1_ and *wt* p53 levels, a direct association was shown between *mt* oncogenic p53 and *hPar1* levels in a panel of prostate cancer cell lines that exhibit low-to-high aggressive properties of prostate cancer with corresponding low-to-high *hPar1* levels, respectively. Both the level of expression and the functionality of PAR_1_ protein were evaluated as indicated by the level of phosphorylated-FAK (e.g., active focal adhesion kinase (FAK)) and transmigration through a matrigel layer. A direct association was demonstrated between the level of *hPar1* expression and *mt* p53, as indicated by temperature sensitive (*ts*) mutants (inactive at 32 °C and active at 37 °C), the level of phospho-FAK, and *hPar1* levels in luciferase activity assays. This outcome provides compelling evidence for the inhibition and delicate regulation of PAR_1_ levels in prostate tumor in the presence of *wt* p53. The presence of *mt* p53 elicits marked levels of oncogenic PAR_1_, suggesting that the *hPar1*-*mt* p53 axis as a target for future therapeutic modalities. 

## 4. Regulation of PAR_1&2_ by Estrogen Response Elements (ERE)

The molecular description of breast tumor subtypes has led to important advancement in treatment. Tumor biopsy specimens that display an estrogen receptor (ER) or a progesterone receptor (PR) respond well to either anti-ER vehicles or aromatase inhibitors, which attenuate estrogen synthesis. HER2-expressing cancers are treated by personalized drugs directed to block HER2 ligand binding and dimerization via antibodies (trastuzumab, pertuzumab), or alternatively by small molecules that inhibit HER2 function such as an HER2 kinase inhibitor (Trikerb). Still, 15–20% of breast cancers fall through the flaws of this system. 

“Triple-negative” breast tumors lack all of the main three molecular signs: ER, PR, and high HER2 expression. Breast cancer expressing ER is controlled by the estrogen (E_2_) hormone, which acts through transcriptional regulation of a panel of target genes. Transcriptional regulation of E_2_ includes the association of E_2_ to ER followed by receptor phosphorylation, receptor dimerization, and binding of the ligand-ER compound to specific motifs, namely, estrogen response elements (ERE) within the promoter of target genes.

Ligation of E_2_ to ER is the driving force in breast tumor development [59,60], serving as a prevailing transcription factor for the enhancement of new genes that play a significant role in physiological and cancer-associated functions [61,62,63,64]. To gain a mechanistic insight to the impact of PAR_1_ in the etiology of breast cancer, we evaluated the functional significance of PAR_1_ levels in clinical tissue microarrays and characterized the biochemistry underlying E_2_ regulation of *hPar1*. The ultimate ongoing challenge is to identify efficient tools to assess the degree of response to a specified therapy. In a 5-year retroactive study of patients with ER-dependent tumors, we found that tumors exhibiting PAR_1_ were associated with considerably shorter disease-free survival (DFS) and shorter overall survival (OS) compared with those that expressed ER but lacked PAR_1_ [14]. A gene signature, such as that obtained by Oncotype Dx (Genomic Health, Inc., Redwood City, CA, USA), for example, provides a gene outline that has been established for ER-positive patients. This outline may be associated with a traditional treatment as well as any necessary refinement, which is part of an oncologist’s practice today [65,66]. For example, a low score may be compatible with hormone treatment, while a high score suggests that additional treatment, such as chemotherapy, might be indicated. ER-ligated transcriptional control of *hPar1* has an aggressive profile in the breast cancer gene imprint. The presence of PAR_1_ may certainly tilt the outcome scoring or stand on its own when making a treatment choice. PAR_1_ classifies a group of patients that need additional therapeutic tools, either chemotherapy or personalized anti-PAR_1_ biological compounds. The detailed outline of E_2_-ER regulation of PAR_1_ as evaluated by ChIP and RT-PCR. Luciferase *hPar1* promoter activity and immunostaining analyses are outlined in detail in Salah Z. et al. [14]. It should be pointed out that in addition to the transcriptional regulation of E_2_-ER it may regulate breast cancer through nontranscriptional regulation recruiting signaling effectors and activating multiple pathways that lead to cellular proliferation [67,68].

## 5. Endocrine Therapy and Selective Estrogen Receptor Modulators (SERMs)

Presently, the treatment approach for hormone-dependent breast tumors is to block the action of E_2_ on cancer cells via one of three approaches: (a) preventing E_2_ from associating with ER with an anti-E_2_, for example tamoxifen [64,65]; (b) inhibiting E_2_ synthesis with an aromatase inhibitor [66]; or (c) downregulation of ER protein levels using an anti-E_2_, for example fulvestrant (faslodex/ICI 182,780) [69]. The most familiar category of therapeutic agents targeting E_2_ action is the selective estrogen receptor modulators (SERMs) for the inhibition and treatment of diseases as osteoporosis and breast tumors [70]. Tamoxifen functions as an E_2_ inhibitor in breast tissue via competitive association to ER, thereby inhibiting an E_2_-stimulated increase in breast tissue cells [71]. Data collected from adjuvant breast cancer trials showed that 5 years of tamoxifen therapy inhibits breast tumor recurrence and diminishes the occurrence of contralateral second primary breast tumors by 50% [72]. Tamoxifen also has advantageous chemopreventive properties and in 1999 it became the first drug accepted by the U.S. Food and Drug Administration (FDA) for breast tumor prevention [73,74]. However, accumulating evidence suggests that resistance can develop, most likely initiated by changes in the ER signal transduction pathway that transforms the inhibitory SERM ERα compound to a progression stimulatory signal [75,76]. The switching of tamoxifen from antagonist to an agonist has been widely examined. It was found that tamoxifen is less powerful in ER-positive breast tumor patients with high expression of HER-2 and the ER coactivator SRC-3 (*AIB1*) [76]. The co-activator *AIB1* (Amplified-in-breast cancer 1) is augmented in ER-positive human breast cancers [77]. *AIB1*, also known as SRC-3 (as well as ACTR, p/CIP, RAC3, TRAM1, and NCOA3), is part of the p160 family, which also includes SRC-1 and SRC-2 [78]. *AIB1* is a transcriptional coactivator that endorses the transcriptional activity of many nuclear receptors such as ER and other transcription factors such as E2F1, AP-1, Sp1 [79,80,81]. ER tasks are mediated primarily by *AIB1* [77,82,83]. *AIB1* overexpression, in combination with high levels of HER-2, was to elicit agonist activity of tamoxifen in experimental cell schemes, and to facilitate resistance to adjuvant tamoxifen treatment [76]. Emerging data suggests that PAR_2_ is the second PAR family member with a major part in breast tumor growth. This is based, among other things, on the detected delay in tumor onset in a murine model for tumor growth following intercross between PAR_2_ knock-out mice and polyoma middle T (PyMT) mice, but not through intercross with PAR1^−/−^ mice [7]. This suggests that PAR_2_ takes a dominant role over PAR_1_ and drives protumor functions. These PAR_2_ protumor functions may be initiated by tissue factor (TF), another coagulation factor, since TF cytoplasmic domain-deleted mice were also shown to have delays in spontaneous breast tumor growth in the polyoma middle T model [84,85]. Accumulating evidence supports the concept that PAR_1_ and PAR_2_ are located in a close proximity and act as one functional unit when establishing heterodimers [86,87]. Consistently, PAR_2_ plays a dominant part in PAR_1_–PAR_2_ instigated tumor activity, since *sh*RNA silencing of *hPar2* effectively inhibits PAR_1_-induced function but silencing *hPar1* does not affect PAR_2_-associated signaling. Accordingly, PAR_2_ represents an attractive therapeutic target in cancer [86]. 

**Figure 2:**
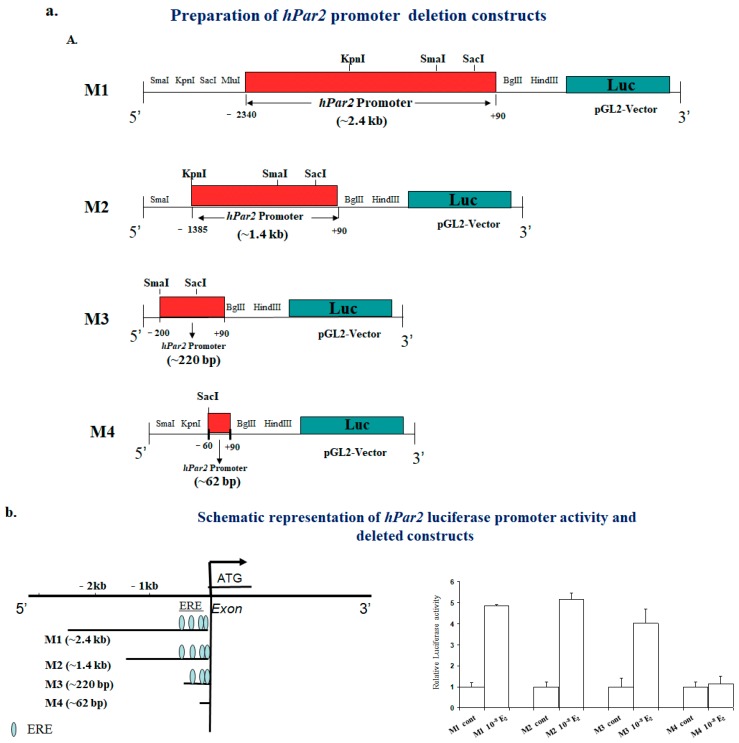

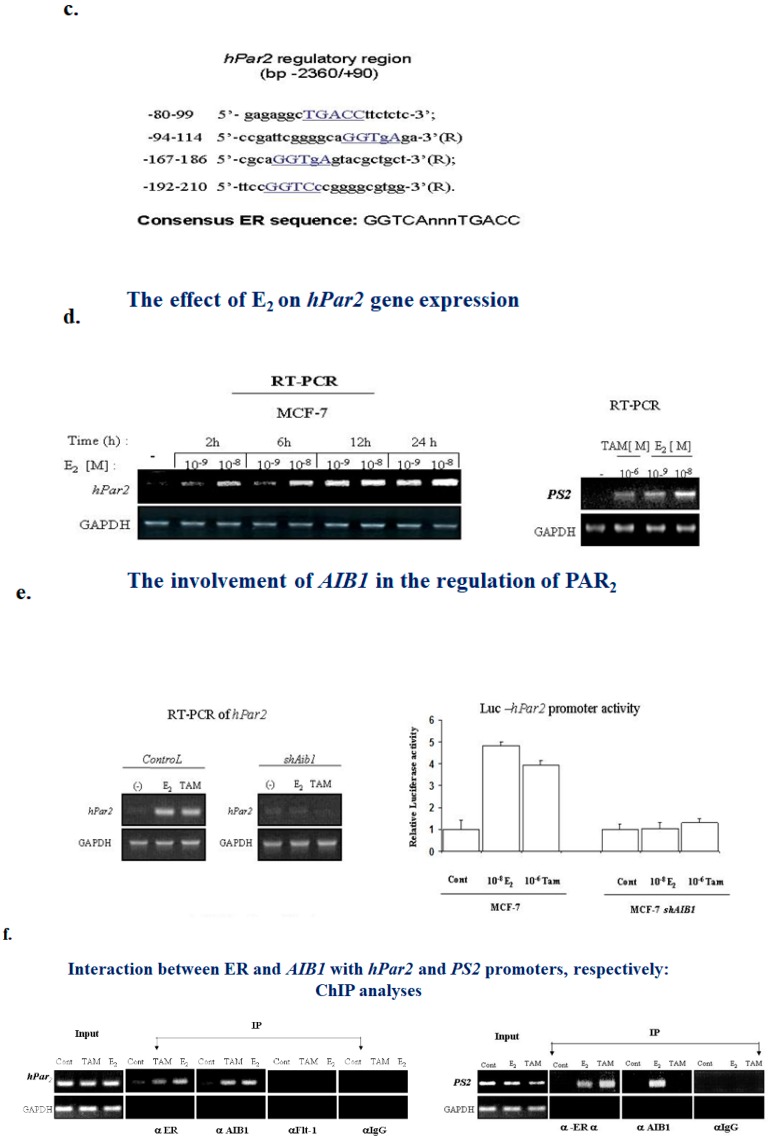

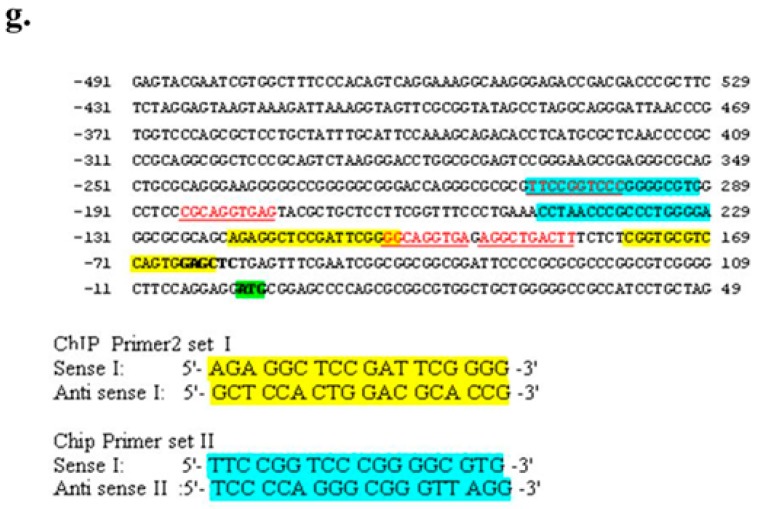
(**a**) Schematic presentation of the *hPar2* promoter cloning and generation of deletion constructs. The *hPar2* promoter was cloned into a pGL2- basic vector. Deleted constructs were generated using an application of appropriate restriction enzymes. The scheme illustrates the various fragments generated. (**b**) Schematic presentation of the *hPar2* promoter and deleted constructs. Luciferase activity of *hPar2* intact promoter and deleted constructs. (**c**) Consensus ER sequence. (**d**) Kinetics of E_2_- or Tam -treated MCF-7 cells. The indicated concentration of E_2_ (10^−8^ M and 10^−9^ M) were applied for various time periods, and the RT-PCR analysis was performed to determine levels of PAR_2_. While a marked enhancement in PAR_2_ level is seen by 2 h of 10^−8^ M treatment and remains till 24 h, no effect is seen by 2 h at 10^−9 M. An elevated level of PAR^_2_ at 10^−9^ M was observed after 6 h of treatment which remained elevated up to 24 h. Dose-response of TAM on the levels of *PS2*, which is a downstream gene target of tamoxifen (TAM). (**e**) Down-regulation of *aib1* inhibited the effect of E_2_ and tamoxifen on *hPar2* expression and proliferation. MCF-7 cells were stably infected with *sh*RNA-*aib1* and maintained for 48 h in phenol red-free medium supplemented with charcoal-stripped fetal bovine serum before either E_2_ or tamoxifen treatment. Then, the medium was changed to a serum-free medium with or without 10^−8^ M E_2_ or tamoxifen at 10^−7^, 10^−6^ M treatment. After two hours, RNA was isolated and RT-PCR analysis of *hPar_2_* was performed. *Sh-aib1* inhibited the effect of tamoxifen and E_2_ induced *hPar_2_-*LUC-promoter activity. MCF-7 *shaib1* cells were transiently transfected with the *hPar2*-LUC reporter construct. After 48 h the cells were treated with 10^−8^ M E_2_ or 10^−7^ and 10^−6^ M tamoxifen for a period of two hours and Luc promoter activity was measured. No effect was observed after E_2_ and tamoxifen treatment. Luciferase activity was normalized to β-gal activity as a control for transfection efficiency. The mean ± standard deviation (SD) are shown (*n* = 6). (**f**) Recruitment of ER to the *hPar2* promoter: ChIP analysis. (**b**) Chromatin fragments immunoprecipitated with the indicated antibodies were purified and the regions containing the ERE-proposed sites were amplified using PCR. An equal amount (input) of DNA was applied. PCR products generated by using either *hPar_2_* promoter primers or GAPDH primers to amplify the immunoprecipitated DNA before and after E_2_ (10^−8^ M) and tamoxifen (10^−6^ M) treatment of MCF-7 are shown. GAPDH primers, control IgG, and a non-relevant (αFlt-1) antibody was used as controls for the evaluation of non-specific immunocomplex formation. Recruitment of ER and *AIB1* to the *PS2* promoter: ChIP analysis. MCF-7 cells were treated with 10^−8^ M E_2_, 10^−6^ M tamoxifen, or with the vehicle alone; chromatin was immunoprecipitated with antibodies against either ER or *AIB1*. The final DNA extracted were amplified using a primer set that covers functional EREs specific to pS2 promoter sequence. Primers specific to unrelated GAPDH gene sequence were used as a control. Input DNA that was amplified by PCR before immunoprecipitation. Control IgG was used as control for non-specific immunocomplex formation. (**g**) 5′-flanking sequences of *hPar2* promoter and proposed *ERE* motifs are shown. EREs are highlighted, along with the sequences of the two sets of primers used.

The presence of functional ERE motifs was shown in the cloned promoter of *hPar2*, with an E_2_-like effect of tamoxifen on *hPar1* and *hPar2* expression (Figure 2) (Jaber M. et al. unpublished data). In addition, the molecular mechanism of tamoxifen-ligated ER involves recruitment of the transcription coactivator *AIB1* to the *hPar1* and *hPar2* promoters. This outcome points to protumor effects of tamoxifen instead of acting as a powerful E_2_-ligated ER antagonist. Upregulation of *hPar1* and *hPar2* by tamoxifen could be responsible, at least in part, for the tumor resistance or even progression seen in a meaningful number of tamoxifen-treated patients. Our findings provide new insights to the progression of breast tumor and endocrine treatment resistance, proposing future approaches for delaying or withdrawing progression by combining tamoxifen treatment with *hPar1* and *hPar2* inhibitors, which are presently reaching clinical trials. Evidence shows that *AIB1* silencing attenuates the effect of E_2_ and tamoxifen on *hPar2* and *hPar1* expression. This was shown through ChIP assays and specific *sh*RNA *aib1* silencing (Figure 2). High *AIB1* expression was shown to modify tamoxifen function from antagonist to agonist on a wide range of target proteins [77,80]. This effect, however, entails both growth factor receptor cross-talk and high *AIB1* protein levels, which lead to ER and *AIB1* phosphorylation [88]. *AIB1* silencing of attenuates the impact of E_2_ or tamoxifen along with TFLLRN or SLIGKV activation of MCF-7 cell-proliferation.

For the purpose of studying E_2_-ligated ER on *hPar2*, we cloned 2400 bp of the promoter region (Jaber M. et al. unpublished data) and prepared deletion constructs of varying length (Figure 2a). This enabled the localization of estrogen response elements (ERE) following bioinformatics search of the *hPar2* promoter. Four ERE candidates within *hPar2* promoter were found, located at: −99–80; 5′-gaga ggcTGACCttctctc-3′; −114–94; 5′-ccgattcggggcaGGTgAga-3′(R); −186–167; 5’-cgcaGGTgAgtac gctgct-3′(R) and −210–192 5′-ttccGGTCccggggcgtgg-3′(R) (Figure 2c). In parallel, two breast tumor cell lines (MCF-7 and T47D) characterized as ER-positive cells that express low-to-no *hPar2* levels and also low-to-no *hPar1* were used. RT-PCR, Western blot and luciferase promoter activity (Figure 2b) were employed to estimate the E_2_ modulation of *hPar2* expression. Increased *hPar2* RNA and protein are seen at 10^−8^ M and also at 10^−9 M E^_2_ (Figure 2d), but was inhibited in the presence of ICI 182,780, a known antagonist of ER. Accordingly, a marked inhibition of the Luc-*hPar2* promoter activity was observed in the presence of ICI (data not shown).

While showing a correlation between *hPar2* expression and ER in breast tumors, we also aimed to assess the effect of tamoxifen on *hPar2* expression. A marked increase in the level of *hPar2* was observed in both RNA and protein in the presence of tamoxifen. To demonstrate *AIB1* involvement in the regulation of *hPar2* by tamoxifen, a stable clone of MCF-7 cells was generated, and it was silenced using *shRNA*-*aib1* following lentiviral infection. MCF-7 cells expressing *sh*RNA for *aib1* were then treated with tamoxifen or by E_2_ and evaluated for *hPar2* level of expression by analysis of RNA levels as well as Luc-promoter activity. A marked inhibition in tamoxifen-treated cells was seen in the presence of *shRNA*-*aib1* (Figure 2e). ChIP analysis was performed on MCF-7 cells before and after E_2_ or tamoxifen therapy. A specific increase in the presence of E_2_ or tamoxifen compared with the untreated cells was observed. By using an antibody towards the irrelevant protein (Flt-1, a cell surface receptor) or control IgG to immunoprecipitate chromatin from the cell lysates (before and after E_2_ usage), negligible levels, which were not affected by E_2_, were seen (Figure 2f,g). When PCR primers for the *pS2* gene (a known estrogen target gene) were utilized in a similar ChIP assay, E_2_ stimulated occupancy of the *pS2* regulatory region by both ER and *AIB1*, while tamoxifen, as assessed, recruited ER, but not *AIB1*, to the *pS2* promoter. These results are in line with the data obtained for *HER2* and ER-*AIB1* [77]. Our results demonstrate a direct binding of ER and *AIB1* to the *hPar2* promoter by tamoxifen. E_2_-ligated ER axis might clarify the agonist function of tamoxifen on the *hPar2* level.

## 6. Regulation by Androgen Response Elements (ARE): Implications to Prostate Cancer

Strongly linked to the tissue context, androgen hormones may instigate the upregulation of a gene imprint, possibly including PAR_1_. Since the androgen hormone drives increases in prostate tumor growth, the standard therapy is androgen ablation. Ablation therapy results in tumor regression up to a point, at which the tumor reappears in an aggressive and androgen-independent form. Androgen receptor (AR) is phosphorylated and forms homodimers upon ligand binding (e.g., testosterone or dihydrotestosterone (DHT)). Ligated AR is translocated to the nucleus, where it functions as a transcription factor through binding to ARE. These motifs are canonical half-site TGTYCT sequences that are separated by three nucleotides from the other half. Nearly no *hPar1* is expressed in normal prostate tissues, whereas high and abundant levels are detected in neoplastic prostate tissue biopsy specimens. The powerful part PAR_1_ plays in prostate cancer progression is shown by comparing radical prostatectomy tissues after androgen ablation with samples taken several weeks prior to the ablation surgery from the same individuals [15]. Apart from *hPar1*, other genes such as FGF8b and VEGF were also shown to be regulated by ligated-AR [15,89,90]. The functional nature of ARE within the *hPar1* promoter was shown using EMSA, Luc-promoter activities, and differential levels of expression in the clinical settings [15]. 

## 7. Twist Transcriptional Regulation

The Twist family of basic helix-loop-helix (bHLH) transcription factors is known to control transcriptional regulation that activates or suppresses the transcription of downstream genes via binding to DNA E-box sequences CAGGTG or CGTCTG [91,92]. Accumulating evidence indicates that PAR_1_ stimulates pro-migratory properties in epithelial malignancies, notably epithelial-mesenchyme transition (EMT), which is characterized by the loss of epithelial indicators (E-cadherin and β-catenins) [92] and the gain of mesenchymal cell markers (fibronectin, vimentin, smooth muscle actin, and N-cadherin). The bioinformatics search for a PAR_1_ promoter pointed to the CAGGTG putative binding motif (unpublished data), although the resulting functionality has not yet been clarified. Twist elicits the expression of PAR_1_ as a downstream gene. This leads to the development of oncogenic traits via inhibition of the Hippo pathway that is induced by PAR_1_ activation [93]. Once Hippo is inhibited, oncogenic YAP/TAZ translocates to the cell nuclei, where it acts as a transcription co-activator of TEAD and induces a pro-tumor gene signature. 

## 8. AP2 and Sp1 Transcription Regulation and Inverse Correlation with Maspin

Transcriptional regulation of *hPar1* in melanoma growth and advancement from the radial to the vertical growth phase has been shown [94]. Whereas an opposite relationship was observed between the high PAR_1_ levels in aggressive melanoma and the activator protein-2 (AP-2), a direct correlation is seen with the expression of specificity protein 1 (Sp1). Analysis of the PAR_1_ regulatory region revealed specific binding motifs for both transcription factors that bind in a mutually exclusive manner for the association motifs located at bp −365 to −329 (complex 1) and bp −206 to 180 (complex 2), depending on the tissue context. It appears that AP-2 is absent in highly aggressive cells while Sp1 takes the lead. In contrast, AP-2 is expressed in the non-metastatic phase whereby Sp1 is absent. It has been proposed that a loss of AP-2 instigates metastatic capability in melanoma [94]. Another inverse correlation in melanoma tissues was also demonstrated between levels of PAR_1_ and Maspin, a tumor suppressor [95]. However, this inverse correlation acts in a different manner, whereby PAR_1_ inhibits binding of Ets-1 and c-Jun transcription factors to the promoter of Maspin. Consequently, PAR_1_ acts indirectly by inhibiting the Maspin tumor suppressor-driven inhibition of melanoma progression and as a result facilitates more aggressive behavior of melanoma cells. 

## 9. TEAD4 and Coactivators YAP/TAZ

The TEA domain (TEAD) family of transcription factors includes four members (TEAD1-4) that control the levels of various genes linked with cell propagation, apoptosis, and differentiation. TEAD4 is the main downstream transcription factor in the Hippo signaling pathway, a major player in tumorigenesis that is often disrupted in tumors. TEAD4 was recently found to be an oncogene and a likely prognostic marker, as well as a therapeutic target in both gastric and breast cancers [96,97]. The transcription coactivators YAP and TAZ, downstream of the Hippo pathway, associate with target genes mostly through their interactions with TEAD4, specifically with the regulatory regions of target genes, via their preserved TEA sites. The Hippo pathway plays a critical part in controlling organ size, and its dysregulation has been connected with numerous tumors [97,98,99]. YAP and TAZ transcription coactivators are the major effectors of the Hippo pathway. Hippo signaling and the YAP/TAZ-TEAD axis are regulated by GPCR signals as well as by multiple factors containing cell–cell interaction and mechanical cues [100,101]. 

Upstream kinases in the mammalian Hippo signaling pathway, including mammalian STE20-like protein kinase 1/2 (MST1/2), activate via phosphorylation of downstream kinases, including large tumor suppressor 1/2 (LATS1/2) with the assistance of the adaptor MOB kinase activator 1A/B (MOB1A/B) and Salvador family WW domain-holding protein 1 (SAV1) [99]. Consecutively, LATS1/2 phosphorylates the final destination YAP/TAZ and causes their anchoring and retention by the cell cytoplasmic pool that serves as a depot reservoir leading to the inhibition of YAP/TAZ. Once de-phosphorylated YAP/TAZ enters the nucleus, the axis functions as an oncogene, interacting and forming a complex with TEADs to regulate the gene signature transcriptome. TEAD proteins are composed of an N-terminal TEA domain and a C-terminal YAP-binding domain (YBD) [102]. The TEAD YBD is the direct link to co-transcription regulatory proteins among which are YAP/TAZ through which the TEA domain is accountable for the association with DNA. The TEA domain of TEADs is greatly preserved throughout evolution and is broadly present in eukaryotes from fungi to mammals [103]. It appears that the TEAD4-regulated transcriptome in colorectal cancer is also rich in genes that contribute to colon cancer recurrence. These observations indicate that TEAD4 is a biomarker for colorectal cancer relapse and plays an important part in the initiation and development of colorectal adenoma.

**Figure 3:**
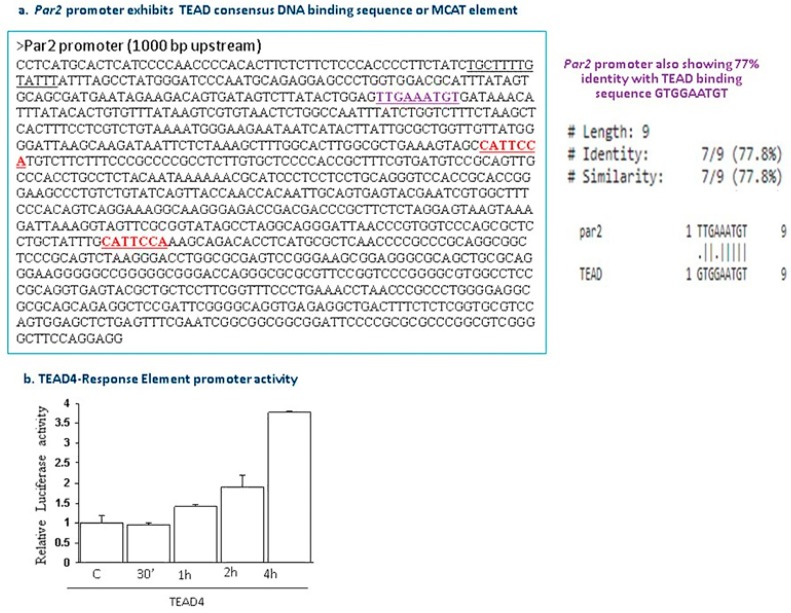
TEAD4 consensus sequence in *hPar2* promoter. (**a**) 5′-flanking sequence of *hPar2* promoter and proposed TEAD4 binding motifs. CATTCCA is the consensus binding motif which is called also M-CAT (M for myfkins family of muscle specific helix-loop-helix family of proteins, followed by the sequence CAT). TTGAAATGT is another sequence found within the promoter of *hPar2* for TEAD binding with 77% homology to GTGGAATGT TEAD binding site. (**b**) TEAD4-LUC promoter activity following SLIGKV activation of PAR_2._

A noticeable task for GPCRs and their associated ligands as controls of Hippo signaling has been recognized [103,104,105,106,107]. The bioactive lipids sphingosine 1-phosphate (S1P) and lysophosphatidic acid (LPA), which act through S1PR and LPA receptors [104,106] via Gα12/13, are powerful inducers of nuclear YAP/TAZ. It has been shown that PAR1 activation potently induces nuclear YAP localization followed by decreased phosphorylation, mediated via Gα12/13 and Rho GTPase [104]. We have identified TEAD4 motifs within *hPar2* promoters 5′-GTGGAATGT-3′ and 5′-CATTCCA-3′ (Jaber M., unpublished data). Indeed, SLIGKV activation of PAR_2_ markedly promoted TEAD4 LUC promoter activity (Figure 3). These outcomes indicate that PARs may serve as potent physiological inducers of the YAP-TEAD4 axis, pointing to a wide range of PAR regulated gene signatures downstream and suggests that they are good targets for therapy. 

## 10. PAR_4_ Transcriptional Regulation

The involvement of PAR_4_ (also known as F2RL3) in cancer is poorly understood, yet it has emerged as a potent stem cell marker among GPCRs for cancer cell sphere formation, as shown in high-throughput RNA sequencing [12]. Recent studies have shown that PAR_4_ is regulated by CpG promoter methylation. PAR4 levels are decreased upon methylation and increased upon hypomethylation in colorectal cancer tissues when compared to matched normal tissues, particularly in poorly differentiated tumors and in lymph nodes with metastases [108]. As a result, transcriptional silencing by promoter hypermethylation has been proposed as a potentially significant tool controlling levels of oncogene expression. It has been shown that 5-Aza-dC, a demethylating agent, restored PAR_4_ levels in the colorectal cancer LoVo cell-line [109]. While DNA methylation is largely considered as a tool for transcriptional suppression, the extent to which it vigorously inhibits transcription factor (TF) binding sites in vivo is not yet known.

DNA methylation plays a major role in imprinting and is necessary for mammalian development [109,110]. Cytosine methylation in the setting of CpG dinucleotides has been broadly considered as a fundamental mechanism for transcriptional suppression at the ATG upstream regulatory regions, and association between DNA methylation and gene expression has long been established [111]. However, the molecular machinery by which DNA methylation disturbs the chromatin state and controls the elements in a site-specific manner remains unclear. The relationship between complete genome DNA methylation and TF binding motifs was studied using the model of TF CCCTC-binding factor (CTCF). An ample TF with recognized methylation sensitivity that is capable of independent association with its target sites in chromatin was assessed [112]. This model is often used to support a major role for CTCF in the organization of chromatin architecture for the whole genome. CTCF is greatly conserved in higher eukaryotes. The full-length protein comprises an eleven zinc finger principal DNA binding site exhibiting close to 100% homology between humans, mice, and chickens. Based on its ability to bind to a spectrum of different sequences as well as specific co-proteins, CTCF was initially defined as a “multivalent factor” [113,114]. These studies showed unequivocally that there is no overall difference in CTCF transcription efficiency with methylated or unmethylated genes. The possibility for DNA methylation to affect TF-binding sets, with a consequent impact on gene expression patterns, has been frequently suggested but not evaluated systematically. Surprisingly, the outcome of these studies is hypothesized to be constant across cellular contexts and under both stable and transient inhibition of DNA methyltransferases, suggesting the effects can be widely generalized. Given the total absence of association between changed binding and methylation variations, these consequences suggest a direct relationship between the level of the CTCF-mediated link between DNA methylation and genome organization. Indeed, a recent study by Yin Y. et al. [115] using a systemic evaluation of 542 TFs showed that some TF, including bHLH, bZIP, and ETS, was inhibited by methylated (mCpG). On the other hand, TFs, such as POU, homeodomain, and NFAT proteins, preferred to bind to methylated DNA. The preference of OCT4, a pluripotency factor of the POU family, to bind to a motif containing mCpG was established and verified by ChIP analysis. Therefore, the language used to read the genome, combined with the basic rules for silencing versus activation instruction, are just starting to evolve and some of the basic instructions are not yet unraveled.

## 11. Gene Expression Profiling of PAR-Related TFs

**Figure 4:**
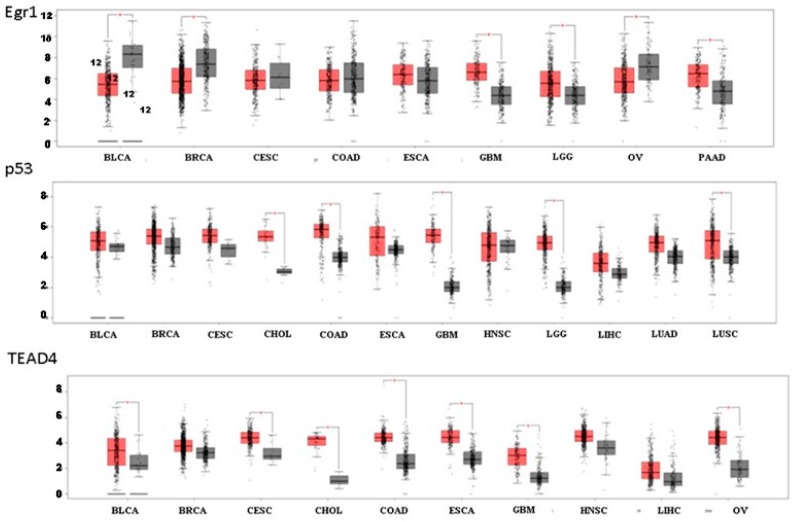
RNA-Seq demonstrating levels of Egr-1, p53, and TEAD4 in different types of epithelial cancers versus healthy individuals using GEPIA analysis. Red box indicates cancer patients and grey box healthy individuals.

**Figure 5:**
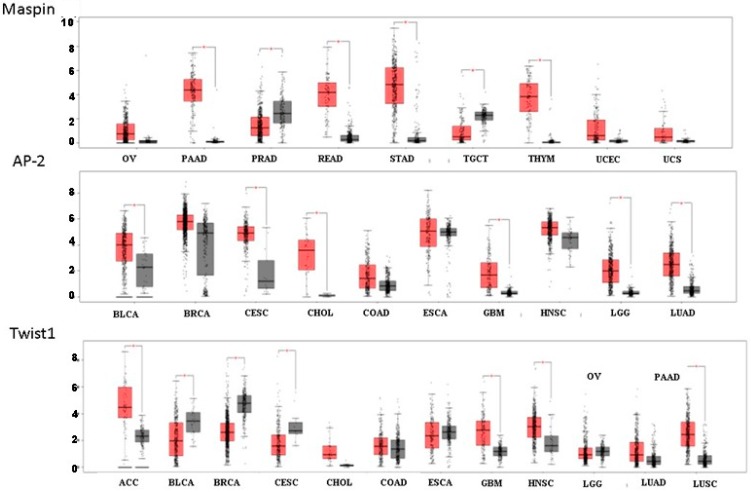
RNA-Seq demonstrating levels of Maspin, AP-2, and Twist1 in different types of epithelial cancers versus healthy individuals using GEPIA analysis. Red box indicates cancer patients and grey box healthy individuals.

It is now possible to perform an efficient online search conducted on a meaningful large cohort of cancer patients versus healthy individuals to assess the level of PAR associated TFs. Such an evaluation may strongly support the importance of the assigned genes and associated transcription factors in the etiology of cancer development. While it has been found that PAR_1_ and PAR_2_ levels are at large upregulated in the majority of epithelial malignancies, both PARs are downregulated in either kidney renal papillary cell carcinoma (for PAR_1_) and kidney chromophobe and kidney chromophobe and skin cutaneous melanoma (for PAR_2_). When we have utilized this online platform using Gene Expression Profiling Interactive Analysis (GEPIA, http://gepia.cancer-pku.cn) ([116]; reviewed by JoAnn Trejo Review in this series), entailing data on RNA sequencing expression, the distinct upregulation of Twist, p53, TEAD, Maspin, and AP2 in the majority of epithelial malignancies was observed (Figure 4 and Figure 5). Unexpectedly, we could not see a pattern of induced levels of Egr-1. In most types of epithelial tumors, equal levels of Egr-1 were obtained in healthy and neoplastic tissues. One should keep in mind that in addition to expression levels (the search is based on RNA-seq), other factors may play an essential part in the final outcome, such as post-translational modifications and cell trafficking that delicately may govern the final outcome of a driver key protein function. The upregulation of the tumor suppressor genes p53 and AP-2 may indicate that their levels are required for the fine-tuning of oncogenes necessary for the promotion of cancer growth and metastasis. 

## References

[1] Dorsam R.T., Gutkind J.S. (2007). G-protein-coupled receptors and cancer. Nat. Rev. Cancer.

[2] Lappano R., Maggiolini M. (2011). G protein-coupled receptors: Novel targets for drug discovery in cancer. Nat. Rev. Drug Discov..

[3] Feigin M.E. (2013). Harnessing the genome for characterization of G-protein coupled receptors in cancer pathogenesis. FEBS J..

[4] Bar-Shavit R., Turm H., Salah Z., Maoz M., Cohen I., Weiss E., Uziely B., Grisaru-Granovsky S. (2011). PAR1 plays a role in epithelial malignancies: Transcriptional regulation and novel signaling pathway. IUBMB Life.

[5] Booden M.A., Eckert L.B., Der C.J., Trejo J. (2004). Persistent signaling by dysregulated thrombin receptor trafficking promotes breast carcinoma cell invasion. Mol. Cell. Biol..

[6] Even-Ram S., Uziely B., Cohen P., Grisaru-Granovsky S., Maoz M., Ginzburg Y., Reich R., Vlodavsky I., Bar-Shavit R. (1998). Thrombin receptor overexpression in malignant and physiological invasion processes. Nat. Med..

[7] Versteeg H.H., Schaffner F., Kerver M., Ellies L.G., Andrade-Gordon P., Mueller B.M., Ruf W. (2008). Protease-activated receptor (PAR) 2, but not PAR1, signaling promotes the development of mammary adenocarcinoma in polyoma middle T mice. Cancer Res..

[8] Kancharla A., Maoz M., Jaber M., Agranovich D., Peretz T., Grisaru-Granovsky S., Uziely B., Bar-Shavit R. (2015). PH motifs in PAR1&2 endow breast cancer growth. Nat. Commun..

[9] Salah Z., Maoz M., Pizov G., Bar-Shavit R. (2007). Transcriptional regulation of human protease-activated receptor 1: A role for the early growth response-1 protein in prostate cancer. Cancer Res..

[10] Yin Y.J., Katz V., Salah Z., Maoz M., Cohen I., Uziely B., Turm H., Grisaru-Granovsky S., Suzuki H., Bar-Shavit R. (2006). Mammary gland tissue targeted overexpression of human protease-activated receptor 1 reveals a novel link to beta-catenin stabilization. Cancer Res..

[11] Nag J.K., Kancharla A., Maoz M., Turm H., Agranovich D., Gupta C.L., Uziely B., Bar-Shavit R. (2017). Low-density lipoprotein receptor-related protein 6 is a novel coreceptor of protease-activated receptor-2 in the dynamics of cancer-associated β-catenin stabilization. Oncotarget.

[12] Choi H.Y., Saha S.K., Kim K., Kim S., Yang G.M., Kim B., Kim J.H., Cho S.G. (2015). G protein-coupled receptors in stem cell maintenance and somatic reprogramming to pluripotent or cancer stem cells. BMB Rep..

[13] Salah Z., Haupt S., Maoz M., Baraz L., Rotter V., Peretz T., Haupt Y., Bar-Shavit R. (2008). p53 controls hPar1 function and expression. Oncogene.

[14] Salah Z., Uziely B., Jaber M., Maoz M., Cohen I., Hamburger T., Maly B., Peretz T., Bar-Shavit R. (2012). Regulation of human protease-activated receptor 1 *(hPar1*) gene expression in breast cancer by estrogen. FASEB J..

[15] Salah Z., Maoz M., Cohen I., Pizov G., Pode D., Runge M.S., Bar-Shavit R. (2005). Identification of a novel functional androgen response element within hPar1 promoter: Implications to prostate cancer progression. FASEB J..

[16] Zhang X., Liu Y. (2003). Suppression of HGF receptor gene expression by oxidative stress is mediated through the interplay between Sp1 and Egr-1. Am. J. Physiol. Renal Physiol..

[17] Nair P., Muthukkumar S., Sells S.F., Han S.S., Sukhatme V.P., Rangnekar V.M. (1997). Early growth response-1-dependent apoptosis is mediated by p53. J. Biol. Chem..

[18] Das A., Chendil D., Dey S., Mohiuddin M., Mohiuddin M., Milbrandt J., Rangnekar V.M., Ahmed M.M. (2001). Ionizing radiation down-regulates p53 protein in primary Egr-1^−/−^ mouse embryonic fibroblast cells causing enhanced resistance to apoptosis. J. Biol. Chem..

[19] Baron V., De Gregorio G., Krones-Herzig A., Virolle T., Calogero A., Urcis R., Mercola D. (2003). Inhibition of Egr-1 expression reverses transformation of prostate cancer cells in vitro and in vivo. Oncogene.

[20] Mora G.R., Olivier K.R., Mitchell R.F., Jenkins R.B., Tindall D.J. (2005). Regulation of expression of the early growth response gene-1 (EGR-1) in malignant and benign cells of the prostate. Prostate.

[21] Yu J., de Belle I., Liang H., Adamson E.D. (2004). Coactivating factors p300 and CBP are transcriptionally cross regulated by Egr1 in prostate cells, leading to divergent responses. Mol. Cell.

[22] Lagos-Quintana M., Rauhut R., Lendeckel W., Tuschl T. (2001). Identification of novel genes coding for small expressed RNAs. Science.

[23] Lau N.C., Lim L.P., Weinstein E.G., Bartel D.P. (2001). An abundant class of tiny RNAs with probable regulatory roles in Caenorhabditis elegans. Science.

[24] Lee R.C., Ambros V. (2001). An extensive class of small RNAs in Caenorhabditis elegans. Science.

[25] Lee Y., Jeon K., Lee J.T., Kim S., Kim V.N. (2002). MicroRNA maturation: Stepwise processing and subcellular localization. EMBO J..

[26] Bartel D.P. (2004). MicroRNAs: Genomics, biogenesis, mechanism, and function. Cell..

[27] Cullen B.R. (2004). Transcription and processing of human microRNA precursors. Mol. Cell.

[28] He L., Hannon G.J. (2004). MicroRNAs: Small RNAs with a big role in gene regulation. Nat. Rev. Genet..

[29] Filipowicz W., Bhattacharyya S.N., Sonenberg N. (2008). Mechanisms of post-transcriptional regulation by microRNAs: Are the answers in sight?. Nat. Rev. Genet..

[30] Siragam V., Rutnam Z.J., Yang W., Fang L., Luo L., Yang X., Li M., Deng Z., Qian J., Peng C. (2012). MicroRNA miR-98 inhibits tumor angiogenesis and invasion by targeting activin receptor-like kinase-4 and matrix metalloproteinase-11. Oncotarget.

[31] Zhang B., Pan X., Cobb G.P., Anderson T.A. (2007). microRNAs as oncogenes and tumor suppressors. Dev. Biol..

[32] Koturbash I., Zemp F.J., Pogribny I., Kovalchuk O. (2011). Small molecules with big effects: The role of the microRNAome in cancer and carcinogenesis. Mutat. Res..

[33] Heintzman N.D., Stuart R.K., Hon G., Fu Y., Ching C.W., Hawkins R.D., Barrera L.O., Van Calcar S., Qu C., Ching K.A. (2007). Distinct and predictive chromatin signatures of transcriptional promoters and enhancers in the human genome. Nat. Genet..

[34] Mito Y., Henikoff J.G., Henikoff S. (2005). Genome-scale profiling of histone H3.3 replacement patterns. Nat. Genet..

[35] Ozsolak F., Song J.S., Liu X.S., Fisher D.E. (2007). High-throughput mapping of the chromatin structure of human promoters. Nat. Biotechnol..

[36] Li D., Ilnytskyy Y., Kovalchuk A., Khachigian L.M., Bronson R.T., Wang B., Kovalchuk O. (2013). Crucial role for early growth response-1 in the transcriptional regulation of miR-20b in breast cancer. Oncotarget.

[37] Wang L., Sun H., Wang X., Hou N., Zhao L., Tong D., He K., Yang Y., Song T., Yang J. (2016). EGR1 mediates miR-203a suppress the hepatocellular carcinoma cells progression by targeting HOXD3 through EGFR signaling pathway. Oncotarget.

[38] Andersson R., Gebhard C., Miguel-Escalada I., Hoof I., Bornholdt J., Boyd M., Chen Y., Zhao X., Schmidl C., Suzuki T. (2014). An atlas of active enhancers across human cell types and tissues. Nature.

[39] Jiao W., Chen Y., Song H., Li D., Mei H., Yang F., Fang E., Wang X., Huang K., Zheng L. (2018). HPSE enhancer RNA promotes cancer progression through driving chromatin looping and regulating hnRNPU/p300/EGR1/HPSE axis. Oncogene.

[40] Shan J., Balasubramanian M.N., Donelan W., Fu L., Hayner J., Lopez M.C., Baker H.V., Kilberg M.S. (2014). A mitogenactivated protein kinase/extracellular signal-regulated kinase kinase (MEK)-dependent transcriptional program controls activation of the early growth response 1 (EGR1) gene during amino acid limitation. J. Biol. Chem..

[41] Parra E., Ortega A., Saenz L. (2009). Down-regulation of Egr-1 by siRNA inhibits growth of human prostate carcinoma cell line PC-3. Oncol. Rep..

[42] Ma J., Ren Z., Ma Y., Xu L., Zhao Y., Zheng C., Fang Y., Xue T., Sun B., Xiao W. (2009). Targeted knockdown of EGR-1 inhibits IL-8 production and IL-8-mediated invasion of prostate cancer cells through suppressing EGR-1/NF-kappaB synergy. J. Biol. Chem..

[43] Yang S.Z., Abdulkadir S.A. (2003). Early growth response gene 1 modulates androgen receptor signaling in prostate carcinoma cells. J. Biol. Chem..

[44] Yang S.Z., Eltoum I.A., Abdulkadir S.A. (2006). Enhanced EGR1 activity promotes the growth of prostate cancer cells in an androgen-depleted environment. J. Cell. Biochem..

[45] Svaren J., Ehrig T., Abdulkadir S.A., Ehrengruber M.U., Watson M.A., Milbrandt J. (2000). EGR1 target genes in prostate carcinoma cells identified by microarray analysis. J. Biol. Chem..

[46] Overholtzer M., Mailleux A.A., Mouneimne G., Normand G., Schnitt S.J., King R.W., Cibas E.S., Brugge J.S. (2007). A nonapoptotic cell death process, entosis, that occurs by cell-in-cell invasion. Cell.

[47] Krishna S., Overholtzer M. (2016). Mechanisms and consequences of entosis. Cell. Mol. Life Sci..

[48] Lang G.A., Iwakuma T., Suh Y.A., Liu G., Rao V.A., Parant J.M., Valentin-Vega Y.A., Terzian T., Caldwell L.C., Strong L.C. (2004). Gain of function of a p53 hot spot mutation in a mouse model of Li-Fraumeni syndrome. Cell.

[49] Olive K.P., Tuveson D.A., Ruhe Z.C., Yin B., Willis N.A., Bronson R.T., Crowley D., Jacks T. (2004). Mutant p53 gain of function in two mouse models of Li-Fraumeni syndrome. Cell.

[50] Liu D.P., Song H., Xu Y. (2010). A common gain of function of p53 cancer mutants in inducing genetic instability. Oncogene.

[51] Joerger A.C., Fersht A.R. (2007). Structure-function-rescue: The diverse nature of common p53 cancer mutants. Oncogene.

[52] Soussi T., Wiman K.G. (2007). Shaping genetic alterations in human cancer: The p53 mutation paradigm. Cancer Cell.

[53] Olivier M., Eeles R., Hollstein M., Khan M.A., Harris C.C., Hainaut P. (2002). The IARC TP53 database: New online mutation analysis and recommendations to users. Hum. Mutat..

[54] Hamroun D., Kato S., Ishioka C., Claustres M., Béroud C., Soussi T. (2006). The UMD TP53 database and website: Update and revisions. Hum. Mutat..

[55] Weisz L., Zalcenstein A., Stambolsky P., Cohen Y., Goldfinger N., Oren M., Rotter V. (2004). Transactivation of the EGR1 gene contributes to mutant p53 gain of function. Cancer Res..

[56] Di Agostino S., Strano S., Emiliozzi V., Zerbini V., Mottolese M., Sacchi A., Blandino G., Piaggio G. (2006). Gain of function of mutant p53: The mutant p53/NF-Y protein complex reveals an aberrant transcriptional mechanism of cell cycle regulation. Cancer Cell.

[57] Aylon Y., Oren M. (2007). Living with p53, dying of p53. Cell.

[58] El-Deiry W.S., Kern S.E., Pietenpol J.A., Kinzler K.W., Vogelstein B. (1992). Definition of a consensus binding site for p53. Nat. Genet..

[59] Lippman M.E., Bolan G.O. (1975). Estrogen-responsive human breast cancer in long-term tissue culture. Nature.

[60] Xu J., Wu R.C., O’Malley B.W. (2009). Normal and cancer-related functions of the p160 steroid receptor co-activator (SRC) family. Nat. Rev. Cancer.

[61] Clarke R.B., Howell A., Potten C.S., Anderson E. (1997). Dissociation between steroid receptor expression and cell proliferation in the human breast. Cancer Res..

[62] McKenna N.J., Lanz R.B., O’Malley B.W. (1999). Nuclear receptor coregulators: Cellular and molecular biology. Endocr. Rev..

[63] McKenna N.J., O’Malley B.W. (2002). Minireview: Nuclear receptor coactivators-an update. Endocrinology.

[64] Paik S., Shak S., Tang G., Kim C., Baker J., Cronin M., Baehner F.L., Walker M.G., Watson D., Park T. (2004). A multigene assay to predict recurrence of tamoxifen treated, node-negative breast cancer. N. Engl. J. Med..

[65] Hortobagyi G.N. (1998). Treatment of breast cancer. N. Engl. J. Med..

[66] Coombes R.C., Gibson L., Hall E., Emson M., Bliss J. (2003). Aromatase inhibitors as adjuvant therapies in patients with breast cancer. J. Steroid Biochem. Mol. Biol..

[67] Giovannelli P., Di Donato M., Giraldi T., Migliaccio A., Castoria G., Auricchio F. (2011). Targeting rapid action of sex steroid receptors in breast and prostate cancers. Front. Biosci..

[68] Castoria G., Migliaccio A., Giovannelli P., Auricchio F. (2010). Cell proliferation regulated by estradiol receptor: Therapeutic implications. Steroids.

[69] Johnston S. (2004). Fulvestrant and the sequential endocrine cascade for advanced breast cancer. Br. J. Cancer.

[70] Jordan V.C. (2004). Selective estrogen receptor modulation: Concept and consequences in cancer. Cancer Cell.

[71] Liu H., Lee E.S., Gajdos C., Pearce S.T., Chen B., Osipo C., Loweth J., McKian K., De Los Reyes A., Wing L. (2003). Apoptotic action of 17 beta-estradiol in raloxifene-resistant MCF-7 cells in vitro and in vivo. J. Natl. Cancer Inst..

[72] Fisher B., Costantino J.P., Wickerham D.L., Redmond C.K., Kavanah M., Cronin W.M., Vogel V., Robidoux A., Dimitrov N., Atkins J. (1998). Tamoxifen for prevention of breast cancer: Report of the National Surgical Adjuvant Breast and Bowel Project P-1 Study. J. Natl. Cancer Inst..

[73] Cuzick J., Powles T., Veronesi U., Forbes J., Edwards R., Ashley S., Boyle P. (2003). Overview of the main outcomes in breast-cancer prevention trials. Lancet.

[74] Johnston S.R., Head J., Pancholi S., Detre S., Martin L.A., Smith I.E., Dowsett M. (2003). Integration of signal transduction inhibitors with endocrine therapy: An approach to overcoming hormone resistance in breast cancer. Clin. Cancer Res..

[75] Nicholson R.I., Gee J.M., Knowlden J., McClelland R., Madden T.A., Barrow D., Hutcheson I. (2003). The biology of anti hormone failure in breast cancer. Breast Cancer Res. Treat..

[76] Osborne C.K., Bardou V., Hopp T.A., Chamness G.C., Hilsenbeck S.G., Fuqua S.A., Wong J., Allred D.C., Clark G.M., Schiff R. (2003). Role of the estrogen receptor coactivator AIB1 (SRC-3) and HER-2/neu in tamoxifen resistance in breast cancer. J. Natl. Cancer Inst..

[77] Anzick S.L., Kononen J., Walker R.L., Azorsa D.O., Tanner M.M., Guan X.Y., Sauter G., Kallioniemi O.P., Trent J.M., Meltzer P.S. (1997). AIB1, a steroid receptor coactivator amplified in breast and ovarian cancer. Science.

[78] Schiff R., Massarweh S., Shou J., Osborne C.K. (2003). Breast cancer endocrine resistance: How growth factor signaling and estrogen receptor coregulators modulate response. Clin. Cancer Res..

[79] Mussi P., Yu C., O’Malley B.W., Xu J. (2006). Stimulation of steroid receptor coactivator-3 (SRC-3) gene overexpression by a positive regulatory loop of E2F1 and SRC-3. Mol. Endocrinol..

[80] Louie M.C., Zou J.X., Rabinovich A., Chen H.W. (2004). ACTR/AIB1 functions as an E2F1 coactivator to promote breast cancer cell proliferation and antiestrogen resistance. Mol. Cell. Biol..

[81] Yan J., Yu C.T., Ozen M., Ittmann M., Tsai S.Y., Tsai M.J. (2006). Steroid receptor coactivator-3 and activator protein-1 coordinately regulate the transcription of components of the insulin-like growth factor/AKT signaling pathway. Cancer Res..

[82] Chen H., Lin R.J., Schiltz R.L., Chakravarti D., Nash A., Nagy L., Privalsky M.L., Nakatani Y., Evans R.M. (1997). Nuclear receptor coactivator ACTR is a novel histone acetyltransferase and forms a multimeric activation complex with P/CAF and CBP/p300. Cell.

[83] Torchia J., Rose D.W., Inostroza J., Kamei Y., Westin S., Glass C.K., Rosenfeld M.G. (1997). The transcriptional co-activator p/CIP binds CBP and mediates nuclear-receptor function. Nature.

[84] Versteeg H.H., Schaffner F., Kerver M., Petersen H.H., Ahamed J., Felding-Habermann B., Takada Y., Mueller B.M., Ruf W. (2008). Inhibition of tissue factor signaling suppresses tumor growth. Blood.

[85] Schaffner F., Versteeg H.H., Schillert A., Yokota N., Petersen L.C., Mueller B.M., Ruf W. (2010). Cooperation of tissue factor cytoplasmic domain and PAR2 signaling in breast cancer development. Blood.

[86] Jaber M., Maoz M., Kancharla A., Agranovich D., Peretz T., Grisaru-Granovsky S., Bar-Shavit R. (2014). Protease-activated-receptor-2 affects protease-activated-receptor-1-driven breast cancer. Cellular and Molecular Life Sciences: CMLS.

[87] Sevigny L.M., Austin K.M., Zhang P., Kasuda S., Koukos G., Sharifi S., Covic L., Kuliopulos A. (2011). Protease-activated receptor-2 modulates protease-activated receptor-1-driven neointimal hyperplasia. Arterioscler. Thromb. Vasc. Biol..

[88] Osborne C.K., Schiff R. (2003). Growth factor receptor cross-talk with estrogen receptor as a mechanism for tamoxifen resistance in breast cancer. Breast.

[89] Ruohola J.K., Viitanen T.P., Valve E.M., Seppänen J.A., Loponen N.T., Keskitalo J.J., Lakkakorpi P.T., Härkönen P.L. (2001). Enhanced invasion and tumor growth of fibroblast growth factor 8b-overexpressing MCF-7 human breast cancer cells. Cancer Res..

[90] Mattila M.M., Ruohola J.K., Valve E.M., Tasanen M.J., Seppänen J.A., Härkönen P.L. (2001). FGF-8b increases angiogenic capacity and tumor growth of androgen regulated S115 breast cancer cells. Oncogene.

[91] Barnes R., Firulli A. (2009). A twist of insight-the role of Twist-family bHLH factors in development. Int. J. Dev. Biol..

[92] Massari M., Murre C. (2000). Helix-loop-helix proteins: Regulators of transcription in eukaryotic organisms. Mol. Cell. Biol..

[93] Wang Y., Liu J., Ying X., Lin P.C., Zhou B.P. (2016). Twist-mediated Epithelial-mesenchymal Transition Promotes Breast Tumor Cell Invasion via Inhibition of Hippo Pathway. Sci. Rep..

[94] Tellez C., McCarty M., Ruiz M., Bar-Eli M. (2003). Loss of activator protein-2 alpha results in overexpression of protease-activated receptor-1 and correlates with the malignant phenotype of human melanoma. J. Biol. Chem..

[95] Villares G.J., Zigler M., Dobroff A.S., Wang H., Song R., Melnikova V.O., Huang L., Braeuer R.R., Bar-Eli M. (2011). Protease activated receptor-1 inhibits the Maspin tumor-suppressor gene to determine the melanoma metastatic phenotype. Proc. Natl. Acad. Sci. USA.

[96] Chan S.W., Lim C.J., Loo L.S., Chong Y.F., Huang C., Hong W. (2009). TEADs mediate nuclear retention of TAZ to promote oncogenic transformation. J. Biol. Chem..

[97] Lim B., Park J.L., Kim H.J., Park Y.K., Kim J.H., Sohn H.A., Noh S.M., Song K.S., Kim W.H., Kim Y.S. (2014). Integrative genomics analysis reveals the multilevel dysregulation and oncogenic characteristics of TEAD4 in gastric cancer. Carcinogenesis.

[98] Yu F.X., Zhao B., Guan K.L. (2015). Hippo pathway in organ size control, tissue homeostasis, and cancer. Cell.

[99] Moroishi T., Hansen C.G., Guan K.L. (2015). The emerging roles of YAP and TAZ in cancer. Nat. Rev. Cancer.

[100] Pan D. (2007). Hippo signaling in organ size control. Genes Dev..

[101] Meng Z., Moroishi T., Guan K.L. (2016). Mechanisms of Hippo pathway regulation. Genes Dev..

[102] Tian W., Yu J., Tomchick D.R., Pan D., Luo X. (2010). Structural and functional analysis of the YAP-binding domain of human TEAD2. Proc. Natl. Acad. Sci. USA.

[103] Burglin T.R. (1991). The TEA domain: A novel, highly conserved DNA-binding motif. Cell.

[104] Yu F.X., Zhao B., Panupinthu N., Jewell J.L., Lian I., Wang L.H., Zhao J., Yuan H., Tumaneng K., Li H. (2012). Regulation of the Hippo-YAP pathway by G-protein-coupled receptor signaling. Cell.

[105] Mo J.S., Yu F.X., Gong R., Brown J.H., Guan K.L. (2012). Regulation of the Hippo-YAP pathway by protease-activated receptors (PARs). Genes Dev..

[106] Miller E., Yang J., DeRan M., Wu C., Su A.I., Bonamy G.M., Liu J., Peters E.C., Wu X. (2012). Identification of serum-derived sphingosine-1-phosphate as a small molecule regulator of YAP. Chem. Biol..

[107] Bao Y., Nakagawa K., Yang Z., Ikeda M., Withanage K., Ishigami-Yuasa M., Okuno Y., Hata S., Nishina H., Hata Y. (2011). A cell-based assay to screen stimulators of the Hippo pathway reveals the inhibitory effect of dobutamine on the YAP-dependent gene transcription. J. Biochem..

[108] Li S.M., Jiang P., Xiang Y., Wang W.W., Zhu Y.C., Feng W.Y., Li S.D., Yu G.Y. (2014). Protease-activated receptor (PAR)1, PAR2 and PAR4 expressions in esophageal squamous cell carcinoma. Dongwuxue Yanjiu.

[109] Yu G., Jiang P., Xiang Y., Zhang Y., Zhu Z., Zhang C., Lee S., Lee W., Zhang Y. (2015). Increased expression of protease-activated receptor 4 and Trefoil factor 2 in human colorectal cancer. PLoS ONE.

[110] Jones P.A. (2012). Functions of DNA methylation: Islands, start sites, gene bodies and beyond. Nat. Rev. Genet..

[111] Jones P.A., Taylor S.M. (1980). Cellular differentiation, cytidine analogs and DNA methylation. Cell.

[112] Deaton A.M., Bird A. (2011). CpG islands and the regulation of transcription. Genes Dev..

[113] Phillips J.E., Corces V.G. (2009). CTCF: Master weaver of the genome. Cell.

[114] Filippova G.N., Fagerlie S., Klenova E.M., Myers C., Dehner Y., Goodwin G., Neiman P.E., Collins S.J., Lobanenkov V.V. (1996). An exceptionally conserved transcriptional repressor, CTCF, employs different combinations of zinc fingers to bind diverged promoter sequences of avian and mammalian c-myc oncogenes. Mol. Cell. Biol..

[115] Yin Y., Morgunova E., Jolma A., Kaasinen E., Sahu B., Khund-Sayeed S., Das P.K., Kivioja T., Dave K., Zhong F. (2017). Impact of cytosine methylation on DNA binding specificities of human transcription factors. Science.

[116] Arakaki A.K.S., Pan W.-A., Trejo J.A. (2019). GPCRs in Cancer: Protease-activated Receptor Expression, Endocytic Adaptors and Signaling. Int. J. Mol. Sci..

